# Lack of Negative Feedback Loops of CTLA-4 and PD-L1 as Key Mechanisms for Post-Acute T Cell Activation Until 3 Months After Ischemic Stroke

**DOI:** 10.3390/ijms262311489

**Published:** 2025-11-27

**Authors:** Lars Peglau, Johanna Ruhnau, Mara Winkelmann, Thomas Krüger, Alexander Dressel, Juliane Schulze, Antje Vogelgesang

**Affiliations:** 1 Department of Neurology, University Medicine, 17475 Greifswald, Germany; 2 Department of Neurology, AMEOS Clinic, 17373 Ueckermünde, Germany; 3 Department of Neurology, Medical University Lausitz-Carl Thiem, 03048 Cottbus, Germany

**Keywords:** CTLA-4, PD-L1, ischemic stroke, T cell activation, microglia, DNT

## Abstract

Post-stroke T lymphocytes exert differential effects both at the infarction site and systemically; these are pro-inflammatory cascades, subsequent T cell infiltration into the brain, and persisting interaction of infiltrating T lymphocytes (more specifically a subset of CD4- CD8-double-negative T cells (DNTs)). Moreover, brain-resident microglia cells exacerbate the parenchymal damage. The acute peripheral immune response is characterized by lymphopenia and persisting activation of circulating T lymphocytes. In the temporal course, it is supposed that anti-inflammatory mechanisms in general and especially the anti-inflammatory M2 phenotype of microglia cells help to recover the functionality of brain parenchyma. We assessed the long-term temporal course of peripheral blood T cell activation post stroke as well as the interaction of pan-T cells or DNTs with microglia cells ex vivo. T cell subpopulations (CD4^+^, CD8^+^, DNT) and their activation (CD25, CD57, CTLA-4 (CD152) and PD-L1 (CD274)) were analysed in peripheral blood from stroke patients and controls by flow cytometry. Pan-T cells and DNTs were isolated by magnetic beads from stroke patients’ blood 3 days (t_1_), 1 month (t_2_), and 3 months (t_3_) post stroke or once from controls, and they were incubated with or without healthy donor human microglia cells ex vivo. Upon interaction, T cell (HLA-DR) and microglia activation (HLA-DR, CX3CR1, CD32, CD86, CD206 and CD209) was assessed by flow cytometry. Moreover, IL-1β in cell culture supernatants was quantified by ELISA. Post-stroke CD4^+^ and CD8^+^ T cell activation (CD25) persisted for 1 month and declined only after 3 months. Inhibitory activation markers CTLA-4 and PD-L1 were upregulated only 3 months after stroke. Co-culture of pan-T cells and microglia had little effect on microglial activation but a strong effect on T cell activation (HLA-DR). Co-culture of DNTs with microglia cells inhibited the M2 phenotype of microglia. Stroke acutely leads to a strong activation (CD25 upregulation) of both CD4^+^ and CD8^+^ T cells. This activation persisted in the subacute post-stroke phase and declined at 3 months post stroke, being accompanied by upregulation of PD-L1 and CTLA-4. Both might be involved in terminating chronic T cell activation. DNTs might influence microglia through CX3CR1 and inhibit an M2 state ex vivo, which might contribute to cerebral inflammation post stroke.

## 1. Introduction

Stroke survivors often suffer from severe long-term disability and cognitive decline [1,2,3]. Thrombolysis and thrombectomy are the most effective therapies in acute ischemic stroke [2,4]. Both therapies restore cerebral blood flow, with the goal of rescuing the perilesional tissue that has not sustained irreversible damage. Such a mismatch can be observed in the early hours following cerebral ischemia, making only a minority of stroke patients eligible for such therapy [2]. Despite clinical improvement due to recanalization, the 90-day mortality of treated and untreated stroke survivors has not improved substantially [5], and 30–50% of patients suffer from cognitive decline, impeding a return to work [6].

It is known that infiltration of immune cells contributes to secondary brain damage; however, so far, studies aiming to improve stroke outcome by modulating immunological or neuroregenerative pathways have not translated into clinical application. As a consequence, a more detailed understanding of the processes in the post-acute stroke phase is required to identify new therapeutic targets.

The immunological consequences of ischemic stroke include both impaired bacterial defense mechanisms facilitating post-stroke infections and excessive activation of cells of the adaptive immune system, including T cells [7]. It is known that T cells are of major importance for functional physiologic cognitive processes [8], and T cell interaction with brain cells and immune cells has been suggested to modulate cognitive recovery after stroke [9,10]. Following ischemic stroke, T cells upregulate activation markers, which are not controlled due to disturbed upregulation of negative feedback loops; one such example is cytotoxic T-lymphocyte-associated protein (CTLA)-4 [7]. Therefore, the activation of T cells persists for several weeks [7,9,11,12,13,14]. Nevertheless, our knowledge of the course of T cell activation in patients post stroke is still very limited: Data are available mainly on CD4^+^ T cells, few activation markers (HLA-DR, CD25, CTLA-4), and for a limited time span of 14 days [7]. While it is undisputed that activated lymphocytes migrate to the ischemic lesion and contribute to secondary lesion growth, detailed knowledge about the role of the immune system during long-term recovery is still scarce. To extend current knowledge beyond the acute and subacute phases, we included a three-month time point representing the early chronic recovery period after stroke. Previous studies have reported persistent lymphocyte activation for several weeks but lack data beyond 14 days [7,11,12]. We therefore aimed to capture the later stage when inhibitory feedback mechanisms, such as CTLA-4 and programmed cell death 1 ligand (PD-L1) expression, may become re-established. The three-month time point represents the post-acute stage of stroke recovery, where sustained systemic immune alterations have been associated with long-term neurological and cognitive outcomes.

The current study was designed to investigate CD25 expression (the alpha chain of the IL-2 receptor) as well as the activation inhibitory feedback loop providers CTLA-4 and PD-L1 on CD4^+^ and the CD8^+^ T cell subgroup. In addition, we focused on the small but functionally relevant double-negative (CD4- CD8-) T cells (DNTs).

Microglia are brain-resident immune cells either shaping a pro- (M1 microglia) or anti-inflammatory (M2 microglia) micro milieu [15,16,17,18]. While the recent literature has rightly criticized the oversimplified M1/M2 dichotomy as unable to capture the full spectrum of microglial phenotypes revealed by modern transcriptomic and proteomic profiling [19,20], we retain this nomenclature here for pragmatic reasons. Despite its limitations, the M1/M2 framework remains a broadly understood and functional shorthand for distinguishing pro- and anti-inflammatory microglial responses in the post-stroke setting, particularly in translational and therapeutic contexts [19,20]. We emphasize that this nomenclature is retained solely as a conventional and practical framework, acknowledging that microglial activation represents a dynamic continuum rather than a strict binary state.

Starting 5 days post stroke, M1 outnumber M2 microglia in the infarction area [16]. M1 microglia downregulate the chemokine receptor (CX3CR)1, which arrests microglia cells in an inactive state upon binding of its ligand CX3CL1 [21]. M1 microglia substantially contribute to persisting neuroinflammation post stroke and produce major proportions of TNF-α in the infarct [22]. By means of a reduced rate of phagocytosis and the inflammatory micromilieu, M1 microglia exacerbate the ramifications of oxygen and glucose deprivation in the infarct. [16,23]. Subsequently, lymphocytes infiltrate the affected brain parenchyma; both CD4^+^ and CD8^+^ T cells migrate to the infarct. By secreting inflammatory Th1 cytokines such as IL-1β and interferon (IFN)-γ, T cells facilitate adhesion of leukocytes and thrombus formation and substantially contribute to neuroinflammation [24]. The resulting elevated IFN-γ concentrations and the correlating temporal course foster the hypothesis that infiltrating T cells shape M1 activation of microglia and maintain their proinflammatory phenotype. So far, this detrimental interaction has only been shown in murine organisms [25].

Within the population of T lymphocytes, CD4- and CD8-double-negative T cells (DNTs) represent a small subset (1.5–7.5%) which exhibit both inflammatory and anti-inflammatory properties [26,27]. Meng et al. observed a significant increase in DNTs in the acute phase post stroke. However, the role of DNTs during stroke-induced immune alteration has not been fully elucidated [28].

To investigate T cell function within the post-acute stroke recovery phase as well as the interaction of T cells, especially that of DNTs with microglia cells, we investigated T cell activation until 3 months post stroke and measured the ex vivo effects of T cells obtained from stroke patients (herein, also DNTs) on human microglia cells and vice versa. In addition, we quantified the release of the proinflammatory cytokine IL-1β by coculturing T cells and microglia cells.

This study addresses the current knowledge gap regarding delayed dysregulation of immune checkpoints after ischemic stroke. We hypothesized that reduced negative feedback via CTLA-4 and PD-L1 contributes to sustained T cell activation three months after the event and may be further modulated by interactions with microglia, including double-negative T cells.

## 2. Results

### 2.1. Activation of T Cellas

In whole blood from stroke patients, the activation markers CD25, CTLA-4, and PD-L1 were quantified on CD4^+^ or CD8^+^ T cells at 3 days (t_1_), 1 month (t_2_), and 3 months (t_3_) post stroke and compared to old as well as young controls for age effects. The percentage of cells expressing the respective activation markers, expression density of markers (MFI) per cell, and absolute cell count per ml blood were determined using flow cytometry.

### 2.2. CD4^+^ T Cell Activation Declined by Month 3 Post Stroke

In the stroke cohort, absolute CD25^+^ CD4^+^ T cell counts were not significantly altered over the study period.

**Figure 1:**
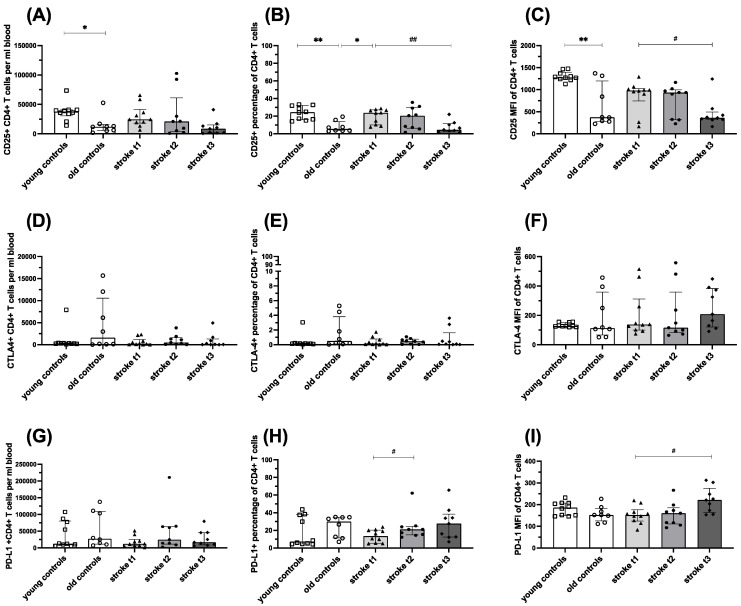
CD25 (**A**–**C**) and CTLA-4 (**D**–**F**) and PD-L1 (**G**–**I**) expression on CD4^+^ T lymphocytes in controls and stroke patients. The percentage of CD4^+^ T cells expressing CD25, CTLA-4, and PD-L1; the amount of these activation markers measured by median fluorescence intensity (MFI); and absolute cell counts per ml blood were quantified from EDTA full blood using flow cytometry. Values are shown as median ± interquartile range, *n* = 10 for “young controls” and “stroke t_1_” groups, *n* = 9 for “stroke t_2_” and “stroke t_3_” groups, *n* = 8 for “old controls” group. Testing was performed for all groups compared with “old controls” and in the stroke cohort between different time points. * ≙ *p* < 0.05 ** ≙ *p* < 0.01 for independent samples; # ≙ *p* < 0.05 ## ≙ *p* < 0.01 for paired samples. Stroke t_1_ (day 3), stroke t_2_ (day 35 ± 2), and stroke t_3_ (day 102 ± 9).

The percentage of CD25-expressing CD4^+^ T cells increased following stroke compared to the age-matched control cohort at one month. At three months, CD25 expression no longer differed significantly from age-matched controls and was significantly reduced compared to one month. Young controls, though, expressed CD25 at levels comparable to acute stroke patients. These findings were robust, as they were seen when the absolute number of CD25-expressing CD4^+^ T cells, the percentage of CD4^+^ T cells expressing CD25, or the level of CD25 expression per cell was analysed. (Figure 1A–C)

In all groups, absolute cell counts and percentages of CTLA-4^+^ CD4^+^ T cells were very low and did not differ significantly between groups. The amount of CTLA-4^+^ on CD4^+^ T cells tended (*p* = 0.26) to be highest in the “stroke t_3_” group (Figure 1D–F).

The absolute cell counts of PD-L1^+^ CD4^+^ T cells did not differ significantly over time post stroke, while the percentage of PD-L1-expressing CD4^+^ T cells increased over time post stroke, being significantly higher at t_2_ than at t_1_. The percentage of PD-L1-expressing CD4^+^ T cells tended (*p* = 0.43) to be lower in young controls than old controls. The amount of PD-L1^+^ in CD4^+^ T cells was highest in the “stroke t_3_” group and significantly higher than in the “stroke t_1_” group (Figure 1G–I).

### 2.3. CD8^+^ T Cell Activation Declined Between One and Three Months Post Stroke

**Figure 2:**
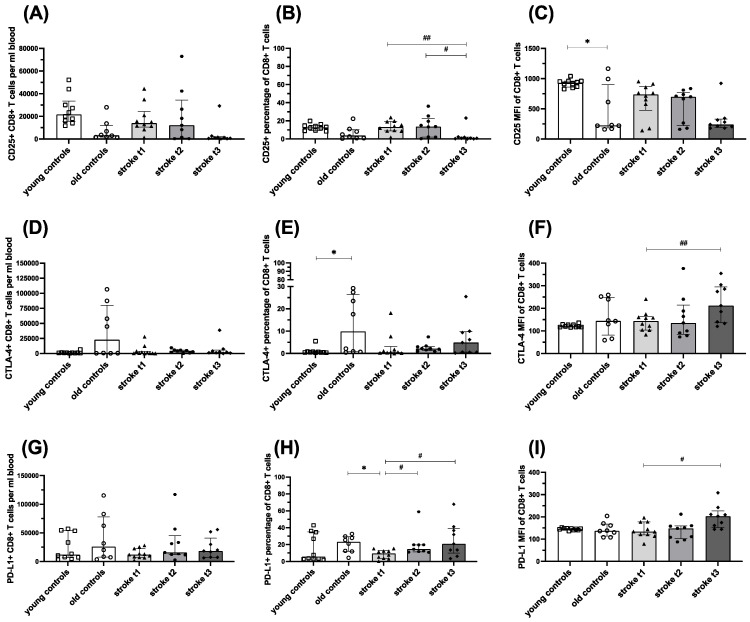
CD25 (**A**–**C**) and CTLA-4 (**D**–**E**) and PD-L1 (**G**–**I**) expression on CD8^+^ T lymphocytes in controls and stroke patients. The percentage of CD8^+^ T cells expressing CD25, CTLA-4 and PD-L1, the amount of these activation markers measured by median fluorescence intensity (MFI) and absolute cell counts per ml blood were quantified from EDTA full blood using flow cytometry. Values are shown as median ± interquartile range, *n* = 10 for group “young controls” and “stroke t_1_”, *n* = 9 for group “stroke t_2_” and “stroke t_3_”, *n* = 8 group “old controls”; testing was performed for all groups compared with „old controls” and in the stroke cohort between different time points. * ≙ *p* < 0.05 for independent samples; # ≙ *p* < 0.05 ## ≙ *p* < 0.01 for paired samples. Stroke t_1_ (day 3), stroke t_2_ (day 35 ± 2) and stroke t_3_ (day 102 ± 9).

Regulation of expression of CD25, CTLA-4 and PD-L1 on CD8^+^ T cells followed the same pattern seen in CD4^+^ T cells. There was a clear trend towards increased activation on “stroke t_1_” and “stroke t_2_” which started to return to CD25 expression seen in age matched controls. This was accompanied by an enhanced expression of the inhibitory molecules CTLA-4 and PD-L1 (Figure 2).

PD-L1^+^ T cells showed very low CD25 expression; analogously CD25^+^ T cells showed very low PD-L1 expression; representative staining is shown in Supplemental Figure S3.

### 2.4. Interaction of Pan-T Cells with Microglia

To assess whether T cells and microglia affect each other, we performed coculture experiments of healthy donor microglia with T cells derived from the peripheral blood of stroke patients at t1, t2, and t3 and from old controls.

### 2.5. Stroke-Derived T Cells Fail to Inhibit HLA-DR Expression on Microglia

**Figure 3:**
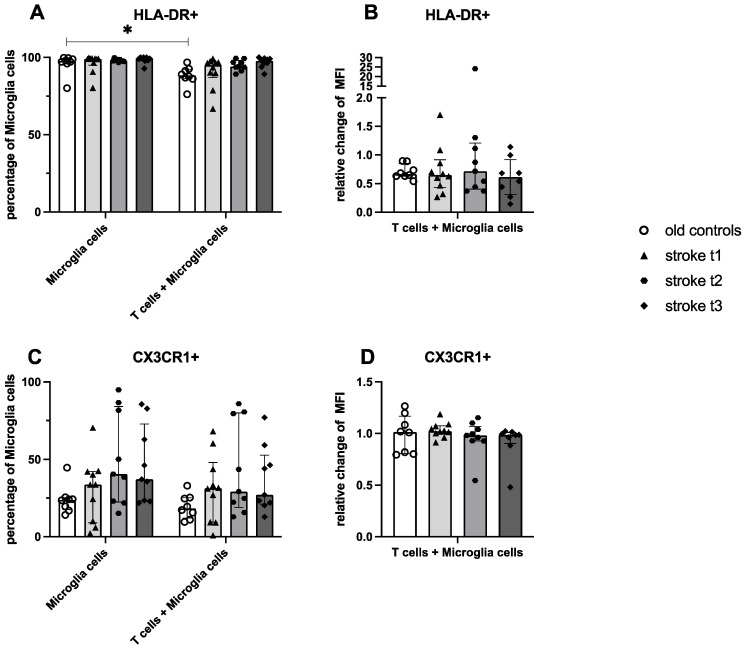
HLA-DR/CX3CR1 expression on microglia cells from controls and stroke patients after 24 h of culture with or without T cells. The percentage of microglia cells expressing HLA-DR and CX3CR1 after 24 h incubation with or without T cells (**A**) + (**C**) and the amount of this activation marker measured by relative change of median fluorescence intensity (MFI) of coculture/monoculture (**B**) + (**D**) were quantified using flow cytometry. Values are shown as median ± interquartile range, *n* = 10 for group “stroke t_1_”, *n* = 9 for group “stroke t_2_” and “stroke t_3_”, *n* = 8 group “old controls”; testing was performed compared with “old controls” and in the stroke cohort between different time points. * ≙ *p* < 0.05 for independent samples. Stroke t_1_ (day 3), stroke t_2_ (day 35 ± 2), and stroke t_3_ (day 102 ± 9).

The activation of microglia from healthy donors was determined after 24 h of incubation in the absence or presence of T cells from stroke patients at the three different time points or from old controls. Expression of HLA-DR and fractalkine receptor CX3CR1 was quantified to determine microglia activation. When cultured alone, a high percentage of microglia cells showed expression of HLA-DR; this expression declined only significantly after coculture with T cells from controls (Figure 3A). The MFI of HLA-DR^+^ microglia did not differ significantly between groups or time points (Figure 3A).

CXCR1 expression on microglia was not significantly altered by coculture with T cells from controls or stroke patients (Figure 3B).

Moreover, T cell coculture did not induce shifts between M1 (CD32 and CD86) and M2 (CD206 and CD209) microglia phenotypes (Figures S5 and S6).

### 2.6. Microglia Cells Induced T Cell Activation Ex Vivo

After 24 h of incubation alone or with healthy donor-derived microglia cells, T cell activation status was analysed based on HLA-DR expression by flow cytometry.

**Figure 4:**
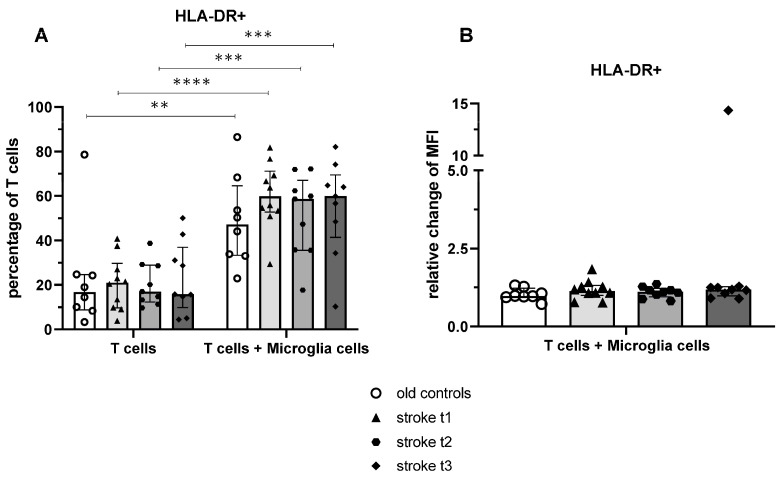
HLA-DR expression on T cells from controls and stroke patients after 24 h of culture with or without microglia cells. The percentage of T cells expressing HLA-DR after 24 h incubation with or without microglia cells (**A**) and the amount of this activation marker measured by relative change of median fluorescence intensity (MFI) of coculture/monoculture (**B**) were quantified using flow cytometry. Values are shown as median ± interquartile range, *n* = 10 for the “stroke t_1_” group, *n* = 9 for the “stroke t_2_” and “stroke t_3_” groups, *n* = 8 for the “old controls” group; testing was performed compared with “old controls” and in the stroke cohort between different time points. ** ≙ *p* < 0.01 *** ≙ *p* < 0.001 **** ≙ *p* < 0.0001 for independent samples. Stroke t_1_ (day 3), stroke t_2_ (day 35 ± 2), and stroke t_3_ (day 102 ± 9).

The average percentage of HLA-DR of T lymphocytes after culture without microglia cells tended (*p* = 0.68) to be lower in the old control group compared to the stroke cohort at t1. (Figure 4A). After coculture with microglia cells, all groups showed a significant increase in HLA-DR expression on T cells, the effect size was lowest for the control group (d_controls_ = 1.26; d_t1_ = 3.06; d_t2_ = 3.02; d_t3_ = 2.65) (Figure 4A).

The relative change in MFI of HLA-DR^+^ T cells was not significant for any comparison (Figure 4B).

### 2.7. Interaction of DNTs and Microglia

**Figure 5:**
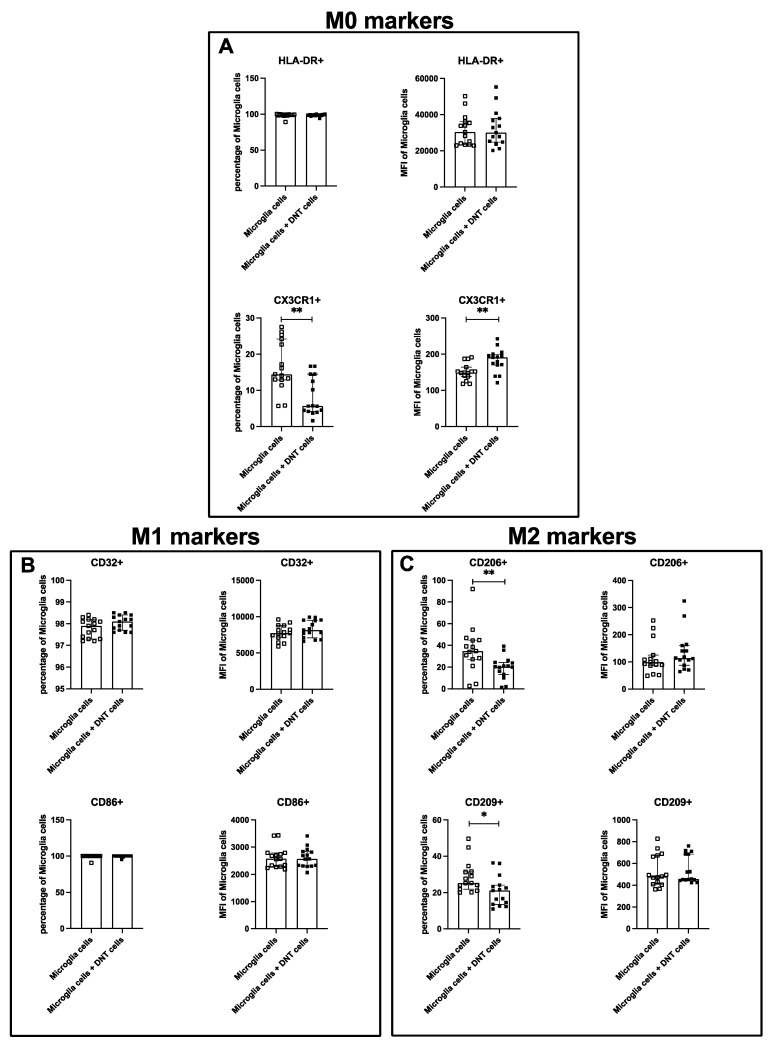
Activation marker expression on microglia cells from controls and stroke patients after 24 h of culture with or without DNT cells. The percentage and MFI of microglia cells expressing HLA-DR and CX3CR1 (**A**), CD32 and CD86 (**B**), and CD206 and CD209 (**C**) after 24 h incubation with or without DNT cells from young healthy donors were quantified using flow cytometry. Values are shown as median ± interquartile range, *n* = 15; testing was performed between culture conditions. * ≙ *p* < 0.05 and ** ≙ *p* < 0.01 for independent samples.

**Figure 6:**
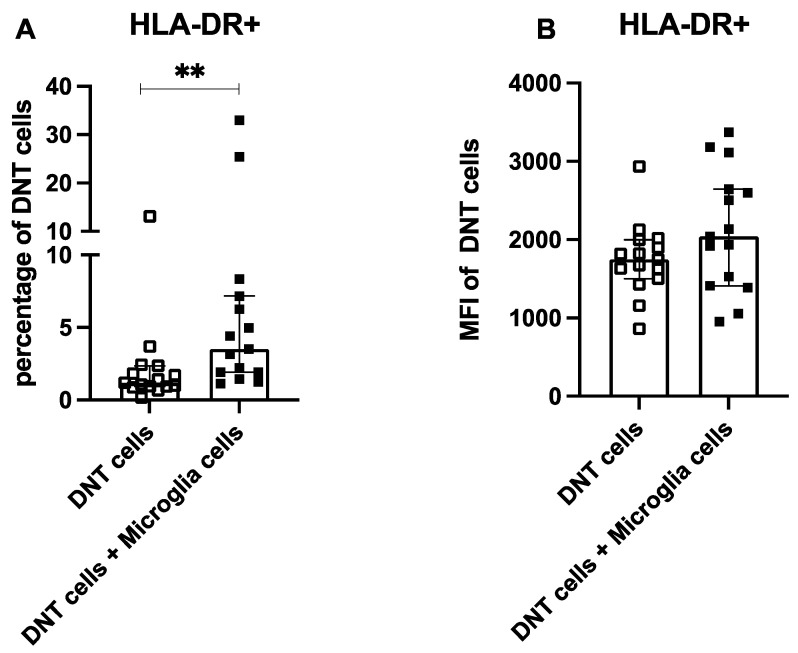
HLA-DR expression on DNT cells from healthy controls after 24 h of culture with or without microglia cells. The percentage of DNT cells expressing HLA-DR after 24 h incubation with or without microglia cells (**A**) and the amount of this activation marker measured by relative change in the median fluorescence intensity (MFI) of coculture/monoculture (**B**) were quantified using flow cytometry. Values are shown as median ± interquartile range, *n* = 15; testing was performed between culture conditions. ** ≙ *p* < 0.01 for independent samples.

Coculture of DNTs showed no significant alteration of HLA-DR for MFI and percentage. Nevertheless, the coculture induced a diminished percentage of CX3CR1, a higher expression of CX3CR1, a reduction of CD206 and CD209 on microglia cells, and increased HLA-DR expression on DNTs (Figure 5). Coculture with microglia cells also significantly increased HLA-DR expression on DNT cells (Figure 6).

### 2.8. IL-1β Concentration After Cell Culture Showed No Effects over Time

**Figure 7:**
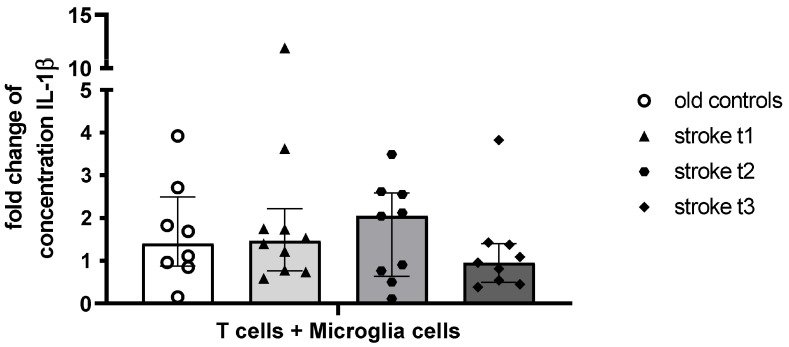
Changes in IL-1β concentration after 24 h of culture of T cells from controls and stroke patients with microglia cells. IL-1β was quantified from cell culture supernatant after 24 h incubation of microglia cells with or without T cells from controls and stroke patients using a high-sensitivity ELISA. The fold change in the concentration IL-1β was calculated as a ratio of concentration (T cells + microglia cells) to concentration (microglia cells).

The fold change in IL-1β concentration quantified by ELISA tended to be highest at t_2_ and lowest at t_3_ in the stroke cohort, but no comparisons of fold changes were significant between the groups (Figure 7).

## 3. Discussion

Stroke alters immune function in several ways. Among them, CD4^+^ T cell activation has been described by several groups within the first weeks after stroke, but it has remained unknown for how long such activation lasts in patients, which pathways could be of importance for chronic activation, and if such activation also applies for CD8^+^ T cells. Extending observation to 3 months post stroke thus enabled assessment of the transition from persistent activation to re-emerging inhibitory signaling through CTLA-4 and PD-L1. Other inhibitory checkpoint pathways such as PD-1, TIM-3, LAG-3, and TIGIT likely contribute to T cell regulation after stroke but were beyond the scope of this focused analysis and should be addressed in future studies.

These observations are consistent with recent reports demonstrating prolonged immune checkpoint alteration and chronic neuroinflammation after stroke in both human and pre-clinical studies [29,30]. While decreased checkpoint expression has been reported in ischemic conditions, the definitive mechanistic pathways through which stroke alters CTLA-4 and PD-L1 signaling remain insufficiently understood. Therefore, our findings should be considered an indication of possible checkpoint dysregulation rather than proof of impaired signaling function.

It is also plausible that sustained T cell activation itself leads to reduced checkpoint expression, indicating a bidirectional relationship rather than a unidirectional failure of inhibition.

This suggests potential implications not only for immune dysregulation but also for cognitive recovery trajectories.

### 3.1. Persistent Activation of CD4^+^ and CD8^+^ T Cells Post Stroke Might Be Terminated via the Late Upregulation of Inhibitory CTLA-4 and PD-L1

In the acute post-stroke phase (t_1_), CD25 expression was significantly increased on both CD4^+^ and CD8^+^ T cells and only declined at t_3_. This expands findings from earlier studies [7] to a period of 3 months and to CD8^+^ T cells. Activation of CD8^+^ T cells has so far only been described in a murine stroke model and only considering the early activation marker CD69 [31]. This chronic T cell activation within the first month is accompanied by a lack of CTLA-4 upregulation, which normally functions as a negative feedback regulator of activation. While at t_3_, the activation of T cells is diminished, CTLA-4 tended to be upregulated again on CD4^+^ T cells and was significantly upregulated on CD8^+^ T cells. The inhibitory activation marker PD-L1 is decreased in the acute post-stroke phase followed by subsequent upregulation. These findings indicate a potential role for PD-L1 and CTLA-4 in the late termination of T cell activation post stroke.

Our findings complement the concept of post-stroke immunosuppression: persistent T cell activation in the peripheral blood can coexist with systemic immune dampening, indicating compartmentalized immune regulation, as suggested by Haeusler et al. (2012) [12]. While altered expression suggests reduced inhibitory feedback, functional assays (e.g., checkpoint blockade or cytokine profiling) would provide stronger causal evidence and are warranted in future studies. Whether persistent T cell activation contributes to secondary complications or poorer functional outcomes after stroke remains to be evaluated in larger, clinically correlated cohorts.

### 3.2. Microglia Cells Activate Both Pan-T Cells and DNTs, but Only DNTs Modulate Microglia Activation Status Ex Vivo

To elucidate potential interactions between microglia and T cells, activation of both cell types after culture alone and coculture was analysed using flow cytometry.

None of the six analysed markers on microglia (HLA-DR, CX3CR1, CD32, CD86, CD206, CD209) showed significant changes in percentage or MFI after coculture with pan-T cells. Thus, pan-T cells from stroke patients were not able to modulate the activation status of healthy microglia ex vivo, in contrast to findings in the murine organism [32,33]. The provided microglia cells showed high percentages of activation markers when incubated alone, which was not described in other studies [34,35]. As a consequence, potential modulatory T cell effects might have been masked by a high degree of pre-activation induced by the isolation procedure performed by the supplier as seen in other studies [36]. In addition, a control experiment for allo-activation was performed (Figure S7). Commercially derived human microglia are known to display partial pre-activation due to isolation-related stress [36]. Resting periods prior to coculture did not further reduce activation levels. While these cells differ from primary microglia in some transcriptomic features [15], they provide a standardized and reproducible human model suitable for ex vivo interaction studies. We have included this limitation explicitly. Since hypoxia is a known activating stimulus for microglia cells [37], we also compared normoxic to hypoxic cell culture conditions in a control experiment but did not find any differences. That said, endotoxicity after stroke causes additional damage-associated molecular patterns which were not included within our ex vivo experiment.

On the other hand, microglia cells effectively activated HLA-DR upregulation on pan-T cells after coculture. This significant effect was not specific for T cells from stroke patients but also observable on T cells from healthy controls; however, the effect size of activation was larger in the stroke cohort.

The absence of statistically significant IL-1β differences likely reflects inter-individual variability and assay sensitivity limits rather than lack of biological effect, as observed effects were concordant with cellular activation markers. Given that other cytokines were below detection, IL-1β served as a feasible indicator of inflammatory activation but warrants confirmation with higher-sensitivity assays in larger cohorts.

T cells and microglia can interact via different pathways either direct cell-to=cell contact or soluble mediators (reviewed in [25]). While Schetters et al. and Chabot et al. showed that Th1 cells were able to induce the inflammatory M1 phenotype, Meng et al. described that not all CD3^+^ T cells (but only the CD4- CD8-DNTs) were colocalized with microglia cells post stroke and were the only T cell subset that polarized microglia activation towards the M1 phenotype [25,28,33]. As a consequence, the coculture setup with pan-T cells might have harbored too few DNTs per well to modulate microglia activation.

We could show that the findings regarding the role of DNTs from murine stroke models [28] coincided with findings using human cells: While HLA-DR did not change after coculture with DNTs, the percentage of CX3CR1 decreased significantly. Since CX3CR1 arrests microglia cells in an idle state and is only downregulated after activation of microglia cells, it is sensible that DNTs activated the microglia cells from the coculture [21]. In our study, CX3CR1 modulation is interpreted as an indirect indicator of microglial activation rather than evidence of direct ligand–receptor signaling, since CX3CL1 concentrations were not measured.

Mechanistically, DNTs may secrete pro-inflammatory mediators such as IFN-γ or IL-17, which promote M1-like microglial activation [28]. This hypothesis aligns with the observed decrease in CX3CR1 and reduction of M2 markers following coculture. Microglia cells increased percentage of HLA-DR on DNTs from coculture significantly, but MFI of HLA-DR did not change significantly.

### 3.3. Limitations

We acknowledge several methodological constraints. Pharmacological treatments such as statins or antihypertensives may have modulated immune activation, although no patient received immunosuppressants. The limited sample size reflects the exploratory design and logistical complexity of longitudinal sampling combined with ex vivo coculture experiments. Effect-size calculations, however, indicated medium-to-large differences between groups, suggesting adequate power for detecting biologically relevant trends. Larger multicenter studies are needed to confirm these observations and assess correlations with clinical parameters.

This study was not capable of sex-specific analyses (43.8% female). As sex hormones influence T cell responses and stroke-induced effects, future studies should address potential sex differences in post-stroke immune activation.

Differences in cardiovascular risk profiles between stroke and control groups may influence baseline immune activation; thus, our results should be interpreted within this clinical context. Further studies will be needed to find whether the results can be found in larger and sex-matched patient cohorts as well.

The use of commercially derived, non-autologous human microglia provides standardized conditions but cannot fully reproduce patient-specific microglial phenotypes or the influence of ischemic priming. This compromise was necessary for ethical and technical reasons yet should be considered when extrapolating to in vivo interactions. Ex vivo coculture systems cannot fully reproduce the hypoxic, vascular, and cytokine gradients of the post-ischemic brain.

Additionally, the cytokine panel was restricted to IL-1β for follow-up analyses, meaning they did not capture the full breadth of inflammatory mediators. Clinical confounders such as comorbidities and ongoing medications might further affect immune marker expression.

The results herein should therefore be interpreted as mechanistic indications rather than direct representations of in vivo dynamics.

## 4. Methods

### 4.1. Patients and Controls

Samples were obtained from a total of 16 patients and 28 controls. Patient and control characteristics are listed in Table 1. Participants included in the individual analysis are stated in the respective figure caption.

Patients were recruited at the Department of Neurology of the University Medicine Greifswald (UMG) as well as Department of Neurology of the AMEOS Clinic Ueckermuende. Inclusion criteria were as follows: National Institute of Health Stroke Scale (NIHSS) score of ≥4; no signs of systemic infection or hyperthyreosis on admission (C-reactive protein (CRP) < 50 mg/L; thyroid-stimulating hormone (TSH) > 0.27 µIU/mL); and written informed consent obtained from the patient or through a surrogate, when applicable. Exclusion criteria were defined as follows: patients receiving immunosuppressive drugs; those diagnosed with a malignancy or autoimmune disease; previous cerebral infarction within the past 6 months; clinically significant anemia; and no written informed consent by the patient themselves or through a surrogate.

To minimize systemic inflammatory confounders, patients with elevated CRP > 50 mg/L, clinical infection, or autoimmune disease were excluded. Most received standard secondary prevention (antihypertensives, statins, platelet inhibitors) but no immunosuppressive medication.

To investigate age-related effects, two control groups were included in addition to stroke patients aged 50 and older. Eight sex- and age-matched control individuals (mean age ± SD: 70.3 ± 9.8 years) were recruited from the ophthalmology clinic, either undergoing treatment for age-related macular degeneration or scheduled for cataract surgery. In addition, 20 young neurologically and immunologically healthy control individuals (mean age ± SD: 23.9 ± 3.2 years) were recruited to specifically examine the influence of age on neurobiological and immunological markers.

### 4.2. Blood Sampling

All venous blood samples were obtained from patients and controls between 7:00 and 10:30 am and processed within 3 h under identical conditions to reduce circadian and pre-analytical variation in immune marker expression.

CRP (C-reactive protein) and thyroid-stimulating hormone (TSH) values (quantified with Dimension Vista, Siemens Healthcare Diagnostics; Eschborn, Germany) were quantified in the central laboratory facility of the UMG. Cell isolation of peripheral blood mononuclear cells (PBMCs) started within 3 h of blood collection.

### 4.3. Pan-T Cell and DNT Isolation

PBMCs were obtained from EDTA-anticoagulated whole blood by separation on a Biocoll density gradient (Biocoll GmbH, Planegg/Martinsried, Germany) using standard centrifugation procedures. Pan-T cells, i.e., all CD3^+^ cells, were magnetically isolated from PBMCs using a Pan-T Cell Isolation Kit (Miltenyi Biotec; Bergisch-Gladbach, Germany) according to the manufacturer’s instructions and resuspended in 1 mL of Microglia Cell Medium (Pelo Biotech; Planegg, Germany).

Where applicable, DNTs were sorted using BD FACSAria^TM^ III (Becton Dickinson and Company; Franklin Lakes, NJ, USA). In total, 360,000 DNTs and 1,000,000 non-DNTs were sorted into two separate tubes. For separation, cells were stained with PE/Cy7 anti-human CD3—Clone: UCHT1, FITC anti-human CD4—Clone: RPA-T4 and PerCP/Cy5.5 anti-human CD8—Clone: SK1 (all BioLegend; San Diego, CA, USA).

### 4.4. Cell Culture

For all cell culture experiments, Costar^®^ 24-well Clear Flat Bottom Ultra-Low-Attachment Multiple-Well Plates (Corning Incorporated; Corning, NY, USA) were used. The used human microglia cells (Pelo Biotech; Planegg, Germany) were stored in liquid nitrogen and thawed in a water bath for 105 s and afterwards counted using a Neubauer chamber. Cell viability (>95%) was verified by trypan blue exclusion and confirmed by flow cytometric gating on live CD11b^+^CD3^−^ cells. The commercial microglia culture (Pelo Biotech) was free of peripheral macrophages or lymphocytes. Thawed cells were adjusted to a concentration of 15,000 cells per ml using Microglia Cell Medium, and 2 mL of cell suspension was added to the respective wells. After incubation at 37 °C and 5% CO_2_ for 8 h, plates were centrifuged (300× *g*, 5 min, Acc. 9, Dec. 7, 37 °C), and 1 mL of old medium was replaced with fresh medium. Plates were then cultured for a further 16 h and centrifuged afterwards (300× *g*, 5 min, Acc. 9, Dec. 7, 37 °C).

For coculture of pan-T cells and DNTs with microglia cells, 1 mL of medium was removed from each well, and 1 mL of pan-T cell or DNT suspension (concentration: 30,000 cells per mL) was added to each well. For only pan-T cell or DNT wells, 2 mL of pan-T cell or DNT suspension (concentration: 30,000 cells per mL) was seeded in empty wells. Microglia cells only served as the control.

### 4.5. Preparation of Whole Blood for Staining

EDTA whole blood was lysed using ACK lysis buffer (1000 mL Aqua destillata—Milli-Q^®^ by Merck; Darmstadt, Germany; plus 8.02 g NH_4_Cl—Carl Roth GmbH; Karlsruhe, Germany; plus 1.0 g KHCO_3_—Merck KGaA; Darmstadt, Germany; plus 0.02 g EDTA—Sigma Aldrich; St. Louis, MO, USA; pH = 7.2) in a 1:10 ratio for 12 min. Lysed blood was centrifuged (300× *g*, 5 min, Acc. 9, Dec. 9, 21 °C) and the supernatant discarded. Cells were washed twice using DPBS (Biochrom GmbH; Berlin, Germany) (300× *g*, 5 min, Acc. 9, Dec. 9, 21 °C).

### 4.6. Analysis of Cell Activation

Cell activation was analysed after 24 h of incubation for cell culture experiments or immediately after peripheral blood withdrawal. Cells from cell culture were harvested using cell scrapers (Greiner Bio One; Kremsmünster, Austria) to regain all cells; cell-free supernatant was collected and stored at −80 °C. Cells were washed twice using DPBS (Biochrom GmbH; Berlin, Germany) (300× *g*, 5 min, Acc. 9, Dec. 9, 21 °C). A Zombie NIR™ Fixable Viability Kit (BioLegend; San Diego, CA, USA) was used for live/dead staining. To prevent nonspecific interactions with Fc receptors, cells were first incubated with a human FcR blocking reagent (Miltenyi Biotec) following the supplier’s recommendations. After this blocking step, surface staining was performed using the corresponding monoclonal antibodies. The panel for surface markers after cell culture included the following: PE/Cy7 anti-human CD3—Clone: UCHT1, BV510 anti-human CD11b—Clone: M1/70, AF488 anti-human HLA-DR—Clone: L243, BV650 anti-human CX3CR1—Clone: 2A9-1, AF647 anti-human CD32—Clone: FUN-2, BV421 anti-human CD86—Clone: IT2.2, PE anti-human CD206—Clone: 15-2, and PerCP/Cy5.5 anti-human CD209—Clone: 9E9A8 (all BioLegend; San Diego, CA, USA). Percentage and median fluorescence intensity (MFI) values were used to quantify marker expression on CD3^−^CD11b^+^ microglia. In parallel, HLA-DR expression was evaluated on CD3^+^ T cells. Fluorescence minus one (FMO) controls were included to verify gating accuracy and exclude spillover-related false positives. Relative changes in MFI were calculated as a quotient of MFI_Microglia_/MFI_coculture_ or MFI_T cells_/MFI_coculture_.

The panel for surface markers for whole blood included the following: FITC anti-human CD4—Clone: RPA-T4, PerCP/Cy5.5 anti-human CD8—Clone: SK1, PE/Cy7 anti-human CD25—Clone: M-A251, PE anti-human CD57—Clone: HCD57, APC anti-human CTLA-4 (CD152)—Clone: L3D10, and PE/Dazzle594 anti-human PD-L1 (CD274)—Clone: 29E.2A3 (all BioLegend; San Diego, CA, USA). Percentage; expression density of activation markers CD25, CTLA-4, and PD-L1 determined by the MFI; and absolute cell counts per ml blood were analysed for CD4^+^ and CD8^+^ T cells. Staining was controlled by using FMO samples for CD25, CTLA-4, and PD-L1.

Data acquisition was performed on an LSR II flow cytometer (BD Biosciences, NY, USA), and subsequent analysis was carried out using FlowJo™ software version 10.5.0 (BD, Ashland, OR, USA). Representative graphs for gating strategy from cell culture and whole blood are shown in the Supplemental Figure (Figures S1 and S2). Further staining controls for expression of T cell activation markers are shown in Supplemental Figures S3 and S4.

All samples were processed using identical instrument settings and compensation controls; nonetheless, inter-day variability cannot be fully excluded.

### 4.7. Analysis of Cytokine Production

We performed an initial screening by cytokine plex (LEGENDplex™ HU Th Cytokine Panel (12-plex), BioLegend, San Diego, CA, USA) within 4 different samples based on where we expected the most effects due to differences observed in cell activation markers. IL-2 (<0.82 pg/mL)), IL-4 (<1.25 pg/mL), IL-5 (<1.44 pg/mL), IL-13 (<0.91 pg/mL), IL-17A (<0.48 pg/mL), IL-17F (<1.17 pg/mL), IL-22 (V1.14 pg/mL), and IFN-g (<1.31 pg/mL) were always below detection limits (see value in brackets). The other cytokines IL-6, IL-9, IL-10, and TNF-a were detectable in some but never in all of the screened samples, revealing rather individual differences between patients than differences based on cell culture conditions. We therefore did not proceed to investigate these prescreened cytokines but continued with IL-1β. IL-1β was selected because of its central role in microglial activation and its reliable detection in preliminary multiplex screening in our cohort.

A high-sensitivity IL-1β ELISA (Thermo Fisher; Darmstadt, Germany) was performed according to the manufacturer’s instructions. Only for the standard curve was a top standard of 20 pg/mL included instead of 10 pg/mL. For analyses, the fold change in IL-1β concentration was calculated as concentration (T cells + Microglia cells)/concentration (Microglia cells).

### 4.8. Statistics

For visualization and statistical analyses, Graphpad Prism 8.2 (GraphPad Software Inc., San Diego, CA, USA) was used. The normality of the data distributions was assessed using the Shapiro–Wilk test. In case of normally distributed data subsets within one analysis, Levene’s test was performed to test for homoscedasticity. To compare more than two groups, a one-way ANOVA or repeated-measures ANOVA was performed in case of homoscedastic data subsets, while the Kruskal–Wallis test or Friedman test was performed in case of heteroscedastic data. Dunn’s post hoc test and Dunnett’s post hoc test was used.

For pairwise comparisons, normality and homoscedasticity were tested as described above. Afterwards, Student’s t-test or the Wilcoxon–Mann–Whitney test were performed. For effect sizes, Cohen’s d was calculated. For all analyses, a *p*-value < 0.05 was regarded as significant.

Given the exploratory nature of this study and the limited cohort size, *p*-values were interpreted descriptively, and effect sizes were reported where appropriate. Formal multiple-comparison corrections were not applied.

## 5. Conclusions

Collectively, our findings suggest a model in which the physiological negative-feedback regulation of post-ischemic T cell activation is weakened. Under normal conditions, engagement of CTLA-4 and PD-L1 limits excessive T cell responses by inhibiting costimulatory signaling and cytokine release. In the post-acute stroke phase, reduced expression of these checkpoints may permit sustained activation and interaction with microglia, contributing to prolonged neuroinflammation. This concept is supported by prior studies linking impaired CTLA-4 or PD-1/PD-L1 signaling to post-stroke immune dysregulation and poor neurological recovery [7,38].

The chronic activation of T cells fades at 3 months post stroke. The lack of negative feedback loops of CTLA-4 and PD-L1 could emerge as key mechanisms and should be studied in future studies including cognitive readouts. DNTs might influence microglia’s activation state through CX3CR1; thus, they inhibit the M2 state ex vivo, which might contribute to cerebral inflammation post-stroke. These mechanisms remain hypothetical, and confirmation in larger longitudinal and functional studies will be required to establish causal links.

The delayed normalization of CTLA-4 and PD-L1 expression may represent a potential therapeutic window within which to restore immune homeostasis after stroke. Pharmacological activation of these checkpoint pathways could theoretically temper chronic neuroinflammation and deserves further preclinical evaluation.

Taken together, these observations suggest that disrupted CTLA-4 and PD-L1 signaling and altered DNT–microglia crosstalk may contribute to persistent T cell activation after stroke. Targeting such pathways could offer novel opportunities for post-stroke immunomodulation, pending validation in future mechanistic studies.

## Figures and Tables

**Table 1:** Cohort characteristics for stroke patients and old and young controls.

Variable	Patient Group (N = 16)	Old Control Group (N = 8)	Young Control Group (N = 20)
Age [Years, Mean ± SD]	68.8 ± 12.6	70.3 ± 9.8	23.9 ± 3.2
Sex [as % female]	43.8	62.5	55.0
**Co-morbidities**			
Hypertension [*n* (%)]	14 (87.5)	2 (25.0)	0 (0.0)
Diabetes mellitus II [*n* (%)]	5 (31.3)	0 (0.0)	0 (0.0)

## Data Availability

The datasets acquired during and/or analysed during the current study are available from the corresponding author upon reasonable request.

## Supplementary Materials

The following supporting information can be downloaded at: https://www.mdpi.com/article/10.3390/ijms262311489/s1.
