# Supplementary material for: Lack of Negative Feedback Loops of CTLA-4 and PD-L1 as Key Mechanisms for Post-Acute T Cell Activation Until 3 Months After Ischemic Stroke
Source: Int J Mol Sci. 2025 Nov 27;26(23):11489. doi: 10.3390/ijms262311489 (PMC12691942; doi:10.3390/ijms262311489)
Supplement: Supplementary file 1 [file ijms-26-11489-s001.zip › ijms-3963674-supplementary.docx]

Supplemental Data

*Supplemental figure S1: Gating strategy for flow cytometry data of T cells from EDTA whole blood.*

Data was plotted Forward-Scatter-Height (FSC-H) / Forward-Scatter-Area (FSC-A) and Sideward-Scatter-Height (SSC-H) / Sideward-Scatter-Area (SSC-A) in order to eliminate doublet cells. Discrimination into dead cells (positive for Zombie stain) and viable cells (Zombie negative/dim) in SSC-A / Zombie plot. Viable cells were plotted SSC-A / FSC-A to select data with Sideward-Scatter and Forward-Scatter values that are characteristic for lymphocytes. In the lymphocyte gate, data was plotted CD4 / CD8 to select CD4+ and CD8+ T lymphocytes. Analyses of activation markers were then performed using FMO samples.

A representative example is depicted. Graphs were created with FlowJo Software. Pseudocolor mode was used to illustrate data point density using a color gradient ranging from blue (very low density) to red (very high density).

**

*Supplemental figure S2: Gating strategy for flow cytometry data of T cells and microglia cells from cell culture.*

Data was plotted Forward-Scatter-Height (FSC-H) / Forward-Scatter-Area (FSC-A) and Sideward-Scatter-Height (SSC-H) / Sideward-Scatter-Area (SSC-A) in order to eliminate doublet cells. Discrimination into dead cells (positive for Zombie stain) and viable cells (Zombie negative/dim) in SSC-A/ Zombie plot. Viable cells were plotted SSC-A/ FSC-A to select data with Sideward-Scatter and Forward-Scatter values that are characteristic for lymphocytes or microglia cells respectively. In the lymphocyte gate, data was plotted SSC-A / CD3 to select CD3+ T lymphocytes. In the microglia cell gate, data was plotted SSC-A / CD3 to select CD3- microglia cells. Analyses of activation markers were then performed using FMO samples.

A representative example is depicted. Graphs were created with FlowJo Software. Pseudocolor mode was used to illustrate data point density using a color gradient ranging from blue (very low density) to red (very high density).

*Supplemental figure S3: Backgating of CD4+CD25+ and CD4+PD-L1+ T cells shows exclusive expression of respective activation markers*

Gating for living single cells was performed as described in Supplemental Figure 1. In the lymphocyte gate, data was plotted CD4/CD8. CD4+ T cells were plotted SSC-A/CD25 or SSC-A/PD-L1 respectively. CD4+CD25+ T cells were plotted SSC-A/PD-L1 and CD4+PD-L1+ T cells were plotted SSC-A/CD25. Gates for activation markers were applied using FMO samples. Graphs were created with FlowJo Software. Pseudocolor mode was used to illustrate data point density using a color gradient ranging from blue (very low density) to red (very high density).

**

*Supplemental figure S4: HLA-DR expression on T cells after culture with or without Microglia cells*

Gating of viable T cells after culture with or without Microglia cells was performed as shown in Supplemental figure 2. T cells were plotted SSC-A/HLA-DR, HLA-DR+ cells were determined using a FMO control. Graphs were created with FlowJo Software. Pseudocolor mode was used to illustrate data point density using a color gradient ranging from blue (very low density) to red (very high density).

*Supplemental figure S5: M1-marker expression on microglia cells after 24 hours of culture with or without T cells from controls and stroke patients.*

The percentage of microglia cells expressing CD32 and CD86 after 24 hours incubation with or without T cells (A,C) and the amount of these activation markers measured by relative change of median fluorescence intensity (MFI) of coculture/pure culture (B,D) were quantified using flow cytometry.

Values are shown as median ± interquartile range, n=10 for group “stroke t_1_”, n=9 for group “stroke t_2_”, n=8 for group “stroke t_3_“ and group “old controls”; testing was performed compared with “old controls“ and in the stroke cohort between different time points. * ≙ p <0,05 ** ≙ p <0,01 *** ≙ p <0,001 for independent samples; # ≙ p <0,05 for paired samples. Stroke t_1_ (day 3), stroke t_2_ (day 35 ± 2) and stroke t_3_ (day 102 ± 9).

*Supplemental figure S6: M2-marker expression on microglia cells after 24 hours of culture with or without T cells from controls and stroke patients.*

The percentage of microglia cells expressing CD206 and CD209 after 24 hours incubation with or without T cells (A,C) and the amount of these activation markers measured by relative change of median fluorescence intensity (MFI) of coculture/pure culture (B,D) were quantified using flow cytometry.

Values are shown as median ± interquartile range, n=10 for group “stroke t_1_”, n=9 for group “stroke t_2_”, n=8 for group “stroke t_3_“ and group “old controls”; testing was performed compared with “old controls“ and in the stroke cohort between different time points. * ≙ p <0,05 ** ≙ p <0,01 *** ≙ p <0,001 for independent samples; # ≙ p <0,05 for paired samples. Stroke t_1_ (day 3), stroke t_2_ (day 35 ± 2) and stroke t_3_ (day 102 ± 9).

*Supplemental figure S7: HLA-DR expression on T cells after 24 hours of culture with or without autologous or allogene monocytes or Microglia cells respectively.*

T cells and monocytes were magnetically isolated from the full blood of healthy, young donors. T cells were then incubated either alone, with autologous monocytes, allogene monocytes or Microglia cells that were commercially available.

The percentage of T cells expressing HLA-DR after 24 hours incubation with or without T cells was quantified using flow cytometry.

Values are shown as median ± interquartile range, n=4 *** ≙ p <0,001.
